# Evidence for Widespread Convergent Evolution around Human Microsatellites

**DOI:** 10.1371/journal.pbio.0020199

**Published:** 2004-08-17

**Authors:** Edward J Vowles, William Amos

**Affiliations:** **1**Department of Zoology, University of CambridgeCambridgeUnited Kingdom

## Abstract

Microsatellites are a major component of the human genome, and their evolution has been much studied. However, the evolution of microsatellite flanking sequences has received less attention, with reports of both high and low mutation rates and of a tendency for microsatellites to cluster. From the human genome we generated a database of many thousands of (AC)*_n_* flanking sequences within which we searched for common characteristics. Sequences flanking microsatellites of similar length show remarkable levels of convergent evolution, indicating shared mutational biases. These biases extend 25–50 bases either side of the microsatellite and may therefore affect more than 30% of the entire genome. To explore the extent and absolute strength of these effects, we quantified the observed convergence. We also compared homologous human and chimpanzee loci to look for evidence of changes in mutation rate around microsatellites. Most models of DNA sequence evolution assume that mutations are independent and occur randomly. Allowances may be made for sites mutating at different rates and for general mutation biases such as the faster rate of transitions over transversions. Our analysis suggests that these models may be inadequate, in that proximity to even very short microsatellites may alter the rate and distribution of mutations that occur. The elevated local mutation rate combined with sequence convergence, both of which we find evidence for, also provide a possible resolution for the apparently contradictory inferences of mutation rates in microsatellite flanking sequences.

## Abstract 2

An analysis of sequences flanking microsatellites in the human and chimpanzee genomes suggests that mutations do not occur independently and randomly, as is commonly assumed in models of DNA sequence evolution

## Introduction

DNA base substitutions do not occur randomly (Graur and Li 2000). Instead, they may be clustered in hotspots, for example around methylated CG dinucleotides, or subject to more general biases such as the excess of transitions relative to transversions. In addition, local structural context may be important, with neighbouring bases interacting to favour some changes over others (Blake et al. 1992; Morton et al. 1997; Goodman and Fygenson 1998; Zavolan and Kepler 2001). However, many nonrandom patterns of sequence evolution remain unexplained. Here we explore how an abundant class of repetitive sequences, microsatellites, may influence the pattern of mutations in sequences that surround them.

Microsatellites are sequences of repeated 1–6-bp motifs that mutate primarily through the gain and loss of repeat units, in a process thought to depend on DNA replication slippage (Levinson and Gutman 1987; Tautz and Schlötterer 1994). Previous studies indicate that their flanking sequences evolve unusually and often contain mutated versions of microsatellites (Matula and Kypr 1999). Estimates of flanking sequence mutation rates vary greatly. Very slow evolution is suggested by sequence comparisons between distantly related species, where divergence rates may be as low as 0.016% to 0.1% per million years (Schlötterer et al. 1991; Rico et al. 1996; Zardoya et al. 1996). Elsewhere, pedigree studies suggest much higher rates and even hypermutability (Stallings 1995). There is also disagreement about trends in mutation rate, some studies indicating an increase towards the microsatellite (Blanquer-Maumont and Crouau-Roy 1995; Zardoya et al. 1996; Grimaldi and Crouau-Roy 1997; Brohede and Ellegren 1999) while others claim a more even distribution (Karhu et al. 2000).

To our knowledge, no one has yet conducted a systematic study of mutational biases operating around microsatellites. The direct study of naturally occurring mutations in flanking sequences is virtually prohibited by their slow rate of accumulation, and inferences based on comparisons between homologous microsatellite loci rely on small numbers of sequences. However, an indirect approach is possible, based on comparisons among very large numbers of microsatellite flanking sequences from the finished human genome. If microsatellites have little or variable influence on their flanking regions, among-locus similarities will be minimal or absent. Conversely, if microsatellites generate similar local mutation biases, nonhomologous loci should betray evidence of convergent evolution. With the publication of large blocks of sequence from the chimpanzee genome, one can extend this approach to ask questions about rate of divergence between homologous flanking sequences.

Here we use a combination of these indirect approaches to show that microsatellites appear to create regions around them in which both the rate and spectrum of mutations are modified.

## Results

**Figure 1:**
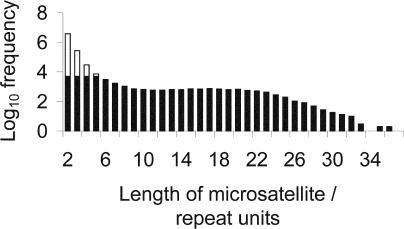
Frequency Distribution of Isolated, Pure (AC)*_n_* Microsatellite Lengths (in Repeat Units) in the Human Genome Black shading indicates numbers of microsatellites used for analyses in this study.

We studied the most abundant class of human dinucleotide repeats, (AC)*_n_,* and for simplicity considered only ‘isolated’ repeats, defined as those at least 100 bp from the nearest AC repeat as small as two units in length. 47% of AC repeats on human Chromosome 1 match these criteria. From the human genomic sequence, maximum sample size was set at 5,000 randomly selected loci for length classes (AC)_2_ to (AC)_5_. For longer microsatellites, of which fewer than 5,000 could be found, all sequences encountered were included. Figure 1 displays the length frequency distribution and sample sizes. Additionally, a control set of 5,000 randomly selected, non-microsatellite-associated sequences, each 50 bases long and containing no (AC)_2+_ repeats, was generated from Chromosome 1.

### Distribution of Cassette Type

**Figure 2:**
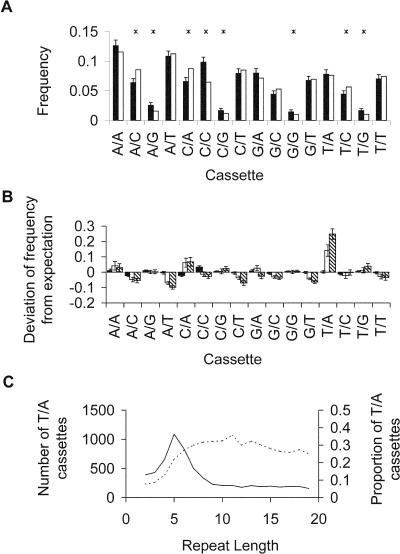
Frequency Distribution of the 16 Possible Microsatellite-Flanking 5′–3′ Base Combinations Relative to Random Expectation (A) Cassette frequencies around (AC)_2_ microsatellites: black bars, observed; white bars, expected. Error bars show 95% confidence intervals and asterisks indicate significant difference (*χ^2^* tests, 1 d.f. *p* < 0.05 with sequential Bonferroni corrections). (B) Deviation of cassette frequencies from random expectations around (AC)_2_, (AC)_5_, and (AC)_10_ microsatellites: black, white, and hatched bars, respectively. (C) Sampled number (solid line) and proportion (dotted line) of microsatellites with cassette T/A as a function of microsatellite length.

To distinguish between (AC)*_n_* and (CA)*_n_* repeats, (AC)*_n_* microsatellites were divided into subclasses, termed ‘cassettes’, according to their immediate 5′ and 3′ flanking bases such that 5′-X(AC)*_n_*Y-3′ is referred to as cassette X/Y. Hence, a (CA)_3_ repeat would be classed as cassette C/A around (AC)_2_. Such a distinction may appear pedantic, but since both DNA replication fidelity and repair efficiency are known to be influenced by base order and local sequence context (Goodman and Fygenson 1998; Marra and Schar 1999), it seems by no means certain that (AC)*_n_* and (CA)*_n_* are equivalent. This nomenclature also helps to resolve the problem of defining microsatellite length because (CA)_3_ equals (AC)_2_. Figure 2 shows the frequency distribution of cassette types relative to expectations, calculated assuming that each cassette base forms a dinucleotide with the end base of the microsatellite it flanks. Thus, cassette X(AC)*_n_*Y is viewed as comprising two dinucleotides, XA and CY. The probability of observing XA and CY jointly is then calculated from the individual frequencies of XA and CY estimated using 1 Mb of randomly sampled sequence from Chromosome 1 containing no (AC)_2+_ repeats.

Around (AC)_2_ microsatellites, the cassette frequencies are broadly similar to those expected from the frequencies of the component dinucleotides in unique sequence DNA, though eight cassettes show significant differences (Figure 2A). However, as AC repeat number increases, the relative frequencies of several cassettes begin to deviate more and more from expectations, either decreasing or increasing in frequency, summarised in Figure 2B. Specifically, cassettes of the kind X/A, and particularly T/A are overrepresented, while cassettes X/C and X/T tend to be underrepresented. The total proportion of (AC) repeats with cassette T/A are shown as a function of repeat length in Figure 2C. The observed pattern could arise either if cassette type influences the rate at which microsatellites change length, or if mutation biases generated by the microsatellite cause interconversion between cassette types.

### Flanking Sequence Base Composition

**Figure 3:**
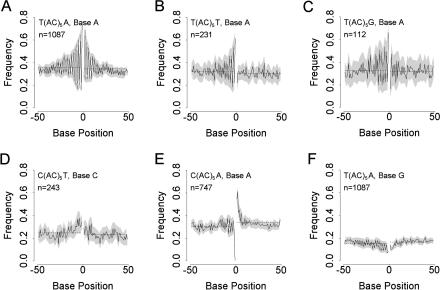
Flanking Sequence Frequency Distributions for Six Representative Nucleotide–Cassette Combinations for (AC)_5_ Microsatellites In each panel, the microsatellite is centrally placed, represented as a gap at position zero, and the cassette type, base, and number of sequences considered *(n)* are given. Frequency distributions are plotted with separate 95% confidence intervals for odd- and even-numbered positions (shading). Horizontal lines indicate mean frequencies for the 3′ and 5′ flanking regions, calculated separately. (A–F) illustrate the six main classes of patterning where either dinucleotide periodicity or 5′–3′ asymmetry are present, summarised for all cassette–base combinations in Table 1.

We consider flanking sequences to extend 50 bases either side of a microsatellite. To compare flanking sequences, we first divided microsatellites according to (AC) repeat number and cassette type, and then calculated the frequency of each of the four nucleotides at each of the 100 possible positions. Any mutation biases present should be revealed by locally changed base composition, and this appears to be the case. For many microsatellite length–cassette combinations, the flanking sequences exhibit strong deviations from random. The observed patterns can be placed in six broad classes according to the strength of a two-base periodicity and the degree of 5′ to 3′ asymmetry. These patterns are illustrated in Figure 3 and summarised in Table 1. Three further classes of less regular patterning can also be defined (Figure S1). For illustration, we chose length (AC)_5_, since this exhibits the strong patterns while at the same time retaining sufficient sample sizes for analyses to be conducted on the rarer cassette types. Several features are apparent. First, levels of patterning can be remarkably strong, with the probability of observing a given base at a given site varying from less than half of that in unique sequence to more than double, often at adjacent sites. Second, many of the patterns show strong dinucleotide periodicities, presumably reflecting the dinucleotide structure of the microsatellite. Third, there are several examples of clear 5′ to 3′ asymmetry, indicating that mutational patterns on one side of a microsatellite may not be the same as those on the other.

**Table 1:**
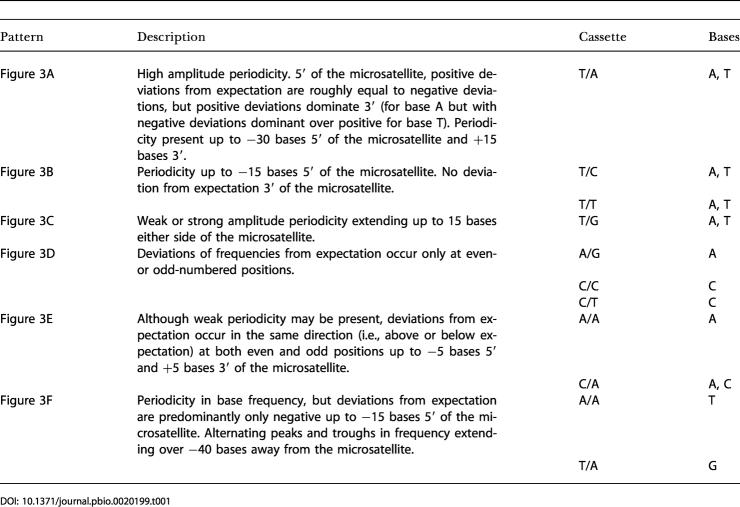
Summary of Patterns in Flanking Sequence Base Frequencies by Cassette

As well as showing variation between cassettes, flanking sequence patterning also changes with microsatellite length. For those cassettes where sample size is sufficient to show a trend, that is, T/A and to some extent C/A and A/A, both amplitude of deviation from random and the breadth of patterning tend to increase with increasing AC repeat number. We do not see dramatic changes such as a reversal in 5′ to 3′ asymmetry or the emergence of new patterns. For cassette T/A, pattern strength increases up to (AC)_9_ but then declines in longer microsatellites. Table 1 reveals a dominant role for nucleotides A and T, with excesses of base A tending to be complemented by deficits at the same positions of base T. In some cases, the excesses of A are interleaved with only weak deviations from base frequency expectation. In other cases, excesses of A are interleaved with excesses of base T.

### Dinucleotide Patterns

**Figure 4:**
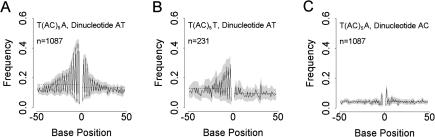
Flanking Sequence Frequency Distributions for Three Representative Dinucleotide Motif–Cassette Combinations for (AC)_5_ Microsatellites (See Figure S4 for the four other patterns). In each panel, the microsatellite is centrally placed, represented as a gap at position zero, and the cassette type, dinucleotide motif, and number of sequences considered *(n)* are given. Frequency distributions are plotted with separate 95% confidence intervals for odd- and even-numbered positions (shading). Horizontal lines indicate mean frequencies for the 3′ and 5′ flanking regions, calculated separately. A summary of how all seven patterns are distributed among all dinucleotide motif–cassette combinations is given in Table 2.

In view of the strong two-base periodicities seen for some base–cassette combinations, we next examined the distribution of frequencies of all 16 possible dinucleotide motifs. As expected from the single nucleotide patterning, dinucleotides also tend to show periodic patterning (Figures 4 and S2), and this is particularly pronounced for motif AT. As with the mononucleotide patterns, there is often marked 5′ to 3′ asymmetry (for example, cassette T/T, dinucleotide AT; Figure 4B). Frequency plots for the 16 cassette types reveal patterns that fall into classes similar to those observed for the mononucleotides, summarised in Table 2. Both the presence of patterning and the degree of 5′ to 3′ asymmetry show a strong dependence on cassette type.

**Table 2:**
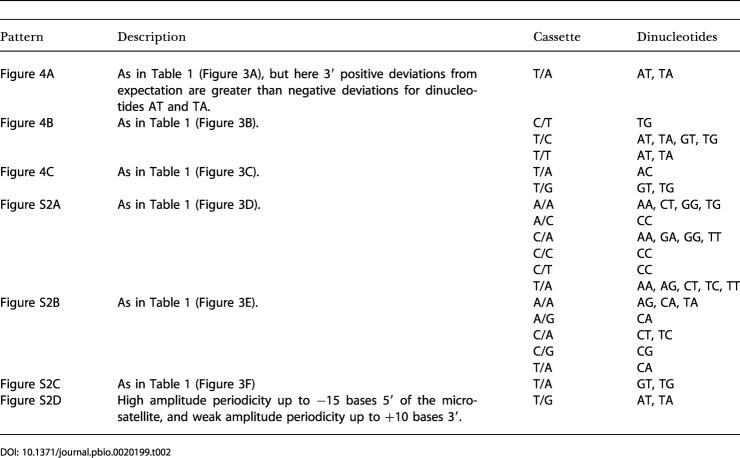
Summary of Patterns in Flanking Sequence Dinucleotide Frequencies by Cassette

From Table 2 it seems that patterning occurs most commonly where the 5′ cassette base is T or the 3′ cassette base is A and least commonly where the 5′ cassette base is either G or A. In almost all cases where patterning is recorded, the dinucleotide involves one or both bases present in the cassette. However, it is unclear whether the flanking pattern is simply an extension of the cassette bases. For example, although 5′ AT or TA periodicity only occurs where the 5′ cassette base is T, CC deviations occur where the 5′ cassette base is A (cassette A/C).

**Figure 5:**
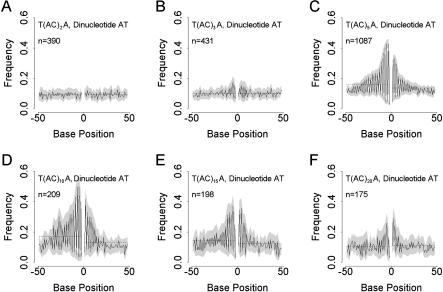
Dependence of Dinucleotide Flanking Sequence Patterning on AC Repeat Number Plots are as described in Figure 4. The progression for dinucleotide AT is illustrated for the commonest cassette type, (T/A). (A–F) depict AT dinucleotide frequencies, where patterning is most extreme, and show how periodicity and amplitude increase towards a maximum at around (AC)_10_ and decline thereafter.

As with the mononucleotide patterning, the periodicity in dinucleotide frequencies changes in amplitude and width (5′ to 3′) as repeat number increases. For most cassettes, the paucity of long microsatellites precludes study of how the patterning changes with repeat number. However, cassette T/A is sufficiently abundant for the progression to be described, and cassettes A/A and C/A, although less common, nonetheless yield meaningful results. Figure 5A–5F shows, for cassette T/A, how the patterning of motif AT first becomes detectable by eye around (AC)_3_, then increases towards a peak in strength at (AC)_9_ before diminishing as AC repeat number increases further. Cassettes A/A and C/A show similar trends and together suggest that, where patterning occurs, it is apparent by (AC)_6_. When data were plotted without first categorizing them by cassette type, although a number of the dinucleotide patterns in Table 1 were seen, deviations from expectation were weaker (for example, about half as strong for dinucleotide AT).

### Other Repetitive Elements in the Genome

An artefactual appearance of convergent evolution could arise if microsatellites within major classes of interspersed repetitive elements such as LINE and Alu repeats are treated as independent observations. Such loci will often share a common origin, and hence appear more similar to each other than expected. To address this problem, we used the program RepeatMasker (Smit and Green 1996) to divide Chromosome 1 into sequences related or not related to known interspersed repeats. Just under half (45%) of all isolated microsatellites were found within interspersed repeats, but only a minority contained microsatellites as long or longer than (AC)_5_. Classifying loci by cassette type, length, and whether they occurred in LINE/L1, SINE/Alu, or unique sequence DNA yielded in most classes sample sizes too low to be of use. However, where sample sizes were adequate, that is, cassette T/A, dinucleotide AT, patterning in unique sequence loci was indistinguishable from that in LINE and SINE microsatellites (Figure S3). If the among-locus similarities were due to shared evolutionary history, we would expect flanking sequences in the three classes to differ. That they do not, suggests an evolutionary process that depends little if at all on original context.

### Microsatellites in the Flanking Sequence

**Figure 6:**
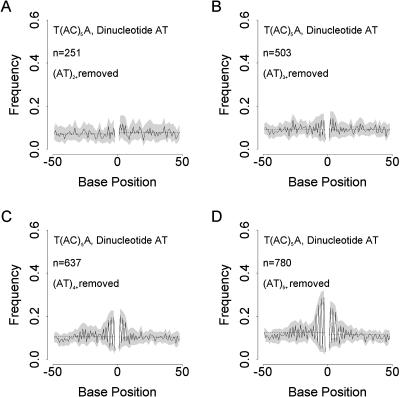
Dependence of Dinucleotide Pattern Strength on the Presence of Repeat Clusters Beginning with the dataset from the scenario showing strong patterning and large sample size (cassette T/A, dinucleotide AT, (AC)_5_; see Figure 5C), flanking sequences containing (AT)*_x_* were excluded, where *x* equalled 2 or more (A), 3 or more (B), 4 or more (C), and 5 or more (D). Plotting conventions are the same as for Figure 4.

The strongest two-base periodicities we find tend to involve motif AT. Such periodicity might arise in two main ways: either because AT motifs in phase with the microsatellite tend to expand through slippage to form (AT)*_n_* microsatellites, or because mutation biases favour the formation of AT motifs in phase with the AC tract. Consequently, we examined the extent to which patterning could be reduced by filtering the flanking sequences for the presence of AT motifs, focusing on cassette T/A and (AC)_5_ to provide strong patterning and large sample sizes. The results of deleting all flanking sequences containing AT microsatellites with *n* or more repeats, where *n* = 2, 3, 4, and 5, are given in Figure 6. This filtering effectively abolishes patterning in all but the region immediately adjacent to the AC repeat tract. Here, patterning extends as far as the maximum value of *n* allowed, suggesting that the dominant AT patterning results from (AT)*_n_* microsatellites developing immediately adjacent to the AC tract.

**Figure 7:**
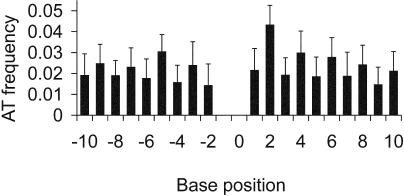
Location of Single AT Dinucleotide Motifs Relative to the Central AC Microsatellite in Flanking Sequences Lacking (AT)_2+_ Microsatellites Figure shows frequency of AT dinucleotides around all length classes of AC repeat microsatellites longer than (AC)_2_ (5′ number of sequences, *n* = 2,924; 3′ number of sequences, *n* = 3,309), with significantly greater numbers at odd positions 5′ and even positions 3′. Data are for cassette T/A only. Error bars show upper 95% confidence limit.

We next examined whether the phase of AT dinucleotides was determined solely by their tendency to form clusters next to (AC)*_n_* repeats. This is an important test given that our strict definition of a microsatellite restricts our analysis to pure AC repeats and allows the possibility that compound repeats, for example, (AT)*_n_*(AC)*_n_*(AT)*_n_* are included. To do this, we plotted the distribution of single AT motifs around (AC)_3+_ microsatellites in the subset of flanking sequences with no (AT)*_n_* microsatellites, where *n* > 1. Summed over all AC microsatellite lengths, single AT dinucleotides are overrepresented in 5′ sequences at odd numbered positions and at even numbered positions in 3′ sequences (Figure 7). Excesses (or deficits) in AT occur up to six bases away from the microsatellites, suggesting that the periodic patterns we see do indeed occur in the flanking sequence over and above the generation of AT microsatellites adjacent to the microsatellite itself. In other words, the patterning we see is generated beyond any tendency for our strict definition of a pure AC repeat to include compound repeats.

### Convergence of Flanking Sequence Pattern

**Figure 8:**
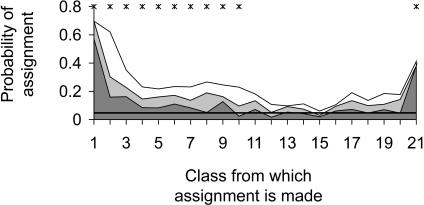
Cross-Locus Similarity among Sequences Flanking Microsatellites of Similar Length Length classes are as follows: class 1, randomly selected sequences not containing (AC)_2+_; classes 2–20, (AC)_2_–(AC)_20_; and class 21, (AC)_21–25_. Figure shows proportion of flanking sequences assigned on the basis of sequence similarity to their own AC repeat number class (dark grey), to the class above (grey), or to the class below (white). Expectation for assignment to self is shown by the horizontal line. Data are for cassette T/A only. Asterisks denote significant overassignment back to the same class or to an adjacent class, tested using *χ^2^* tests (*p* < 0.05 using sequential Bonferroni corrections).

To assess the level of any convergent evolution, we used a simple assignment test to determine how often individual sequences resemble others flanking unrelated microsatellites of similar length (see Materials and Methods). Figure 8 summarises these results. If all sequences were evolving divergently, any given sequence would be assigned to each of the microsatellite length classes with equal probability of around 5%. Sequences not associated with (AC)_2_ or longer were assigned back to their own class 57% of the time, showing that these sequences consistently differ from those near to AC repeat tracts. Remarkably, this figure falls to almost half (32%) for sequences flanking (AC)_2_, indicating that, even with just two repeats, similarities to other microsatellite flanking sequences already exist. The same pattern extends to other length microsatellites, with flanking sequences tending to be preferentially assigned back to their own or to an adjacent length class. When a flanking sequence is not assigned back to its own class, it is usually assigned to one of three other classes: the ‘1’ class of random sequences, the ‘9’ class where patterning is strongest, or to the longest class, class 21 (unpublished data).

**Figure 9:**
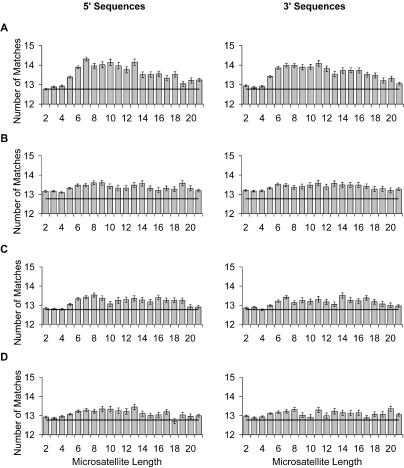
Dependence of Sequence Similarity among Flanking Sequences on AC Repeat Number The average number of matches shown (± standard error) quantifies similarity among three classes of sequence: (1) blocks of 50 bp lying immediately adjacent to a microsatellite; (2) blocks of 50 bp chosen randomly to lie between 500 and 600 bases downstream from a microsatellite; and (3) randomly selected blocks of 50 bp from around the genome. Average level of chance similarity in the genome is shown by a black line in each plot (comparison among class 3). 5′ and 3′ sequences are shown separately. Comparisons among sequence classes are shown for class 1 to class 1 (A), class 1 to class 2 for sequences at the same locus (B), class 1 to class 2 for sequences at different loci (C), and class 1 to class 3 (D).

These analyses reveal a tendency for microsatellite flanking sequences to be similar to each other, but fail to quantify the level of sequence change involved. To do this, we sought to estimate similarity among three classes of sequence: (1) blocks of 50 bp lying immediately adjacent to a microsatellite; (2) blocks of 50 bp chosen randomly to lie between 500 and 600 bases downstream from a microsatellite (the random selection aims to remove possible complications of exact position and phase with the microsatellite); and (3) blocks of 50 bp randomly selected from around the genome. Comparisons within class 3 define the average level chance similarity in the genome, here estimated at 12.77 ± 3.28 (sd) bases out of 50. Comparisons within class 1 estimate how much convergent evolution is apparent at any given repeat number, and reveal a profile that rises to a maximum of 14.31 at a length of seven repeats, followed by a gentle decline with increasing length thereafter (Figure 9A). Similarity is significantly above random for all but the very shortest microsatellites. As controls, we also made comparisons between class 1 and class 2 within a locus (Figure 9B), between class 1 and class 2 among loci (Figure 9C), and between class 1 and class 3 (Figure 9D). Each of these comparisons reveals above random similarity in a profile that approximates that of the class 1–class 1 comparisons but peaking at lower levels.

Thus, in all cases, sequences immediately flanking a microsatellite show greater similarity to each other, to sequences nearby, and to sequences elsewhere in the genome than randomly selected sequences do to each other, a trend that is maximal for microsatellites around 7–10 repeats in length. We believe these similarities are generated primarily by the enhanced simplicity of microsatellite flanking regions and their tendency to gain AT motifs. Such characteristics allow unusually high matches when compared with random blocks of 50 bases that have high simplicity or contain polyA tracts. Over and above this background level of elevated similarity, proximity to any microsatellite appears to increase similarity, implying that microsatellites tend to lie more generally in regions of similar base composition. This might reflect either a tendency for microsatellites to arise preferentially in certain broad sequence contexts, or modification of the local base composition by the microsatellite itself. Moreover, similarity is further enhanced when a flanking sequence is compared with a neighbouring block. This suggests a local context effect such as might arise through the isochore structure of the genome, with neighbouring blocks being located in the same isochore and hence ‘coloured’ by the same nucleotide biases.

### How Big Is the Sphere of Influence of a Microsatellite?

**Figure 10:**
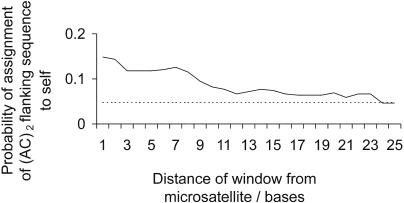
Relationship between the Probability of Assigning (AC)_2_ Microsatellite Flanking Sequences to Self and Proximity to the AC Microsatellite Solid line shows the probability of assignment back to self. Analysis is restricted to (AC)_2_ flanking sequences and is based on an assignment window 25 nucleotides wide on each side of the microsatellite. Dotted line indicates assignment probability expected of random DNA sequences.

To define more precisely the regions where convergent evolution is occurring, we repeated the assignment test but instead of using the full 50 bases either side we now used a symmetric pair of moving 25-base windows placed either side of the AC microsatellite (Figure 10). Close to the microsatellite, the assignment probability is similar to but a little greater than that observed for the full 50-base analysis. As expected, this value declines as the window is moved away from the microsatellite. However, overassignment of (AC)_2_ flanking sequences to their own class is significant up to ten bases away from the microsatellite (*χ^2^* = 9.7, d.f. = 1, *p* < 0.05 with sequential Bonferroni correction), and only when the window reaches 24 bases from the microsatellite does the assignment level fall to the value of 4.8% expected of random sequences.

### Do Microsatellites Increase the Local Rate of Evolution?

So far we have considered only the nature of the mutations that affect flanking sequences, and not their rate. To examine whether mutation rates are affected by the presence of a microsatellite, we used Megablast (National Center for Biotechnology Information (NCBI); ftp://ftp.ncbi.nih.gov/blast/executables) to compare microsatellites identified in completed sections of the chimpanzee genome against homologous loci identified in humans. If homologous loci are identified through comparisons among their immediate flanking sequences, an element of circularity will be introduced, since loci with lower rates of evolution will be identified preferentially. To circumvent this problem, we used a region 300 bases in length and 220 bases downstream of the microsatellite to conduct each Megablast search.

A total of 8218 chimpanzee loci were identified, and they yielded 5537 unique human homologues. Since microsatellite flanking sequences may contain insertions or deletions, we adopted the following approach so as to minimise problems of alignment. The chimpanzee flanking sequence was divided into 20 contiguous blocks of 20 bases, ten on each side of the microsatellite. Each block was then compared against 1,000 bases of human sequence, downstream from the region identified by Megablast, to find the best possible match. To filter sequences with major rearrangement we required both that all 20 blocks match with at least 15/20 bases, and that matched blocks all lie in the same order as their homologues. Differences between pairs of homologous flanking sequences were then quantified, at each of the 20-block positions, as the proportion of perfectly matching blocks and the average number of matches within each block (Figure S4).

After the above filtering, our data base contained 5017 sequences (91% of the original number), and among these, approximately 77% of the blocks were identical. Around (AC)_2–3_ there is little apparent variation in either measure of similarity with block position apart from a small tendency for 5′ blocks to show an increasing average percentage match closer to the microsatellite. However, although the trends are by no means strong, around longer microsatellites similarity of blocks near to the microsatellite is reduced, particularly when measured using average percentage matches (Figure S4C and S4D).

Our analysis of dinucleotide frequencies around microsatellites shows patterns of similarity on a smaller scale than might be revealed with 20-base blocks of sequence. To investigate the number of changes in the immediate flanking bases, we also calculated the proportion of mismatches occurring at a given base for each 5′ and 3′ sequence in the immediate flanking blocks (blocks −1 and +1; Figure S5). The average proportion of mismatches is relatively constant along the flanking sequence around short microsatellites, with a possible rise immediately 3′ of the microsatellites. However, the clear overabundance of mutations in the immediate flanking region of long microsatellites, with a higher than average proportion of mismatches −9 bases 5′ and +4 bases 3′, indicates that the regions closest to the microsatellites are indeed experiencing elevated mutation rates. The dearth of mismatches further away from the microsatellites, and a similar overall mean proportion of mismatches to that around short microsatellites (0.012 versus 0.011, respectively), suggests that a higher mutation rate close to microsatellites is occurring at the expense of the number of mutations further from microsatellites.

## Discussion

We have studied very large numbers of (AC)*_n_* microsatellite flanking regions culled from the human genome and asked questions about the extent that these evolve in any consistent and unusual manner. Patterning is present in the form of over- and underrepresentation of bases and dinucleotide motifs at odd and even positions either side of the microsatellite. Pattern strength is maximal around (AC)_9_, but appears present even around sequences as short as (AC)_2_ and may extend as many as 50 bases either side. Some patterning is more or less symmetrical, but we also found several examples showing strong 5′ to 3′ asymmetry, implying that the two ends of a microsatellite are by no means equivalent. The net result is that sequences flanking microsatellites of a given length tend to be more similar to each other than to random sequences or to sequences flanking microsatellites of different lengths. Thus, there appears to be convergent evolution. Finally, we compared large numbers of homologous flanking sequences between humans and chimpanzees, and found evidence that mutation rates near microsatellites tend to be somewhat elevated.

Sequences surrounding (AC)*_n_* tracts exhibit remarkable levels of patterning, with any given dinucleotide motif tending to be much more likely to occur at even numbered positions rather than odd, or vice versa. For several reasons, we believe that the patterning arises due to the structural properties of the microsatellite (see below), becoming more pronounced as repeat number increases. These reasons include the following: the consistently central placement of microsatellites within the patterning, the dependence of the strength of patterning on AC repeat number, the similarity between microsatellites in LINE and SINE elements and those elsewhere, the weakness of the patterning around (AC)_2_, and the strong influence of cassette type on the form of patterning. Unfortunately, it is surprisingly difficult to eliminate the alternative hypothesis, namely that the patterning arises due to some other force and that AC repeats then either form or expand more rapidly when placed centrally within the pattern. This ambiguity is particularly relevant to the question of cassette distribution, where it seems reasonable both that (AC)*_n_* tracts might cause biased interconversion between cassettes and that certain cassettes may allow slippage more than others. For example, while the structural properties of AC repeats are known to generate mutational biases in adjacent bases (Timsit 1999) capable of changing cassette type, minisatellite mutation rate can depend critically on the presence of a particular base in the flanking sequence (Monckton et al. 1994).

The relationship between an AC microsatellite and its flanking sequences begins surprisingly early, with (AC)_2_ already showing a small but significant bias in the distribution of cassette types and greater similarity to other sequences flanking AC microsatellites than to random sequences. In addition, the moving window assignment test indicates that significant convergence exists even when the ten bases closest to the microsatellite are excluded. Such a wide influence around such a common, short motif is remarkable and suggests that a high proportion of the genome may be affected by these and similar forces. To illustrate, (AC)_2_ is expected to occur every 250 bases, as is (GT)_2_. Taking the sphere of influence on each side as ten bases plus half the 25-bp window yields a value of 45. This predicts that over 30% (approximately 45 bases of every 125) of the genome will be affected by (AC)_2_ on one strand or the other, a figure that will only increase with inclusion of longer arrays and other microsatellite motifs.

As AC repeat number increases, so does the strength of patterning, becoming pronounced by (AC)_5_ and peaking in strength at (AC)_9_. Although patterning is seen in several different dinucleotide motifs, even in the human genome there are insufficient data to study any but the commonest cassette–motif combinations over a wide range of microsatellite lengths. Focusing on the motif AT, we found evidence that the strongest patterning was due to the development of AT microsatellites abutting AC tracts. However, this is not the only effect. After removal of all (AT)_2_ or longer microsatellites, there remains a significant tendency for single AT motifs to appear in phase with AC tracts, suggesting that mutation bias as well as slippage is involved.

Given the increase in strength of patterning between (AC)_2_ and (AC)_9_, it might seem logical that the pattern would become stronger and stronger as repeat number increases further. Instead, (AC)_9_ appears to be the peak strength, with longer microsatellites showing lower amplitude but a broader spread of patterning. It is interesting that this peak coincides with the length at which microsatellites begin to become polymorphic: a common rule of thumb for marker development in mammals is that primers are designed for loci carrying ten or more repeats (Weber 1990). This may be mere coincidence or may reflect, for example, a change in mutation process associated with individuals who are heterozygous for alleles carrying different repeat numbers (Rubinsztein et al. 1995; Amos et al. 1996; Amos and Harwood 1998). Again, there are parallels with minisatellites, where many mutations occur by the transfer of material from one homologous chromosome to the other (Jeffreys et al. 1995).

The rich patterning we find presumably arises through local mutation biases. Previous work on mutation biases has tended to reveal either generic effects such as isochors (Bernardi 2000), where some bases are favoured over others in large regions of the genome, or specific but highly localised biases where one or two bases may influence what happens to their immediate neighbours (Blake et al. 1992; Morton et al. 1997; Goodman and Fygenson 1998; Zavolan and Kepler 2001). The patterns we find suggest a somewhat intermediate process in which mutational dependency appears to extend over distances of 30 bases or more. At the same time, the patterning is position dependent, in that it involves not just, for example, a favouring of A over other bases, but, instead, a favouring of A over other bases at even numbered sites.

The actual mechanism that causes patterning remains unclear, but our data suggest a model based on the structural properties of AC repeat tracts. Local variation in DNA structure is known to be associated with mutational biases (Morton et al. 1997) and variation in mutation rate (Petruska and Goodman 1985; Goodman and Fygenson 1998), as well as possibly influencing the mismatch repair process (Werntges et al. 1986; Marra and Schar 1999). Tracts of repeating AC motifs tend to exhibit unusual structural properties with high propeller twist and shifted base pairing (Timsit 1999), and hence may be considered prime candidates for sequences capable of influencing the evolution of their immediate surroundings. Indeed, crystallographic studies indicate that sequences like (AC)*_n_* and (A)*_n_* induce local mutation biases (Timsit 1999).

The unusual structure of microsatellite DNA may generate mutational biases in at least two ways. First, in AC repeat tracts, each base interacts unusually strongly with the neighbour of its complement base in a way that may lead to misincorporation of incoming nucleotides toward the ends of the microsatellite or in the immediate flanking region. Second, AC tract structure may influence the efficiency of the mismatch repair machinery in correcting either noncomplementary bases or loops resulting from slipped strand misalignment of repetitive DNA. Given that the mismatch repair system is strongly implicated in moderating the otherwise high rates of slippage mutation at microsatellite loci (Levinson and Gutman 1987; Schlötterer 2000), it seems possible that even a small bias in the repair of loop structures might be responsible for the patterning we observe. However, although variation in mismatch repair efficiency may depend to some extent on DNA structure, the effect of sequence context on repair is not well understood (Marra and Schar 1999). Unfortunately, with current understanding, none of these mechanisms would generate mutation biases that extend tens of bases away from the microsatellite, and hence this aspect must await further research.

An alternative explanation for some of the patterning, for example, the tendency for single AT motifs to lie in phase with the microsatellite, could be that these elements represent the remnants of a longer and now eroded (AC)*_n_* repeat tract. Under this scenario, point mutations at specific positions along the microsatellite would presumably interrupt the repeats. Given a strong bias toward transition mutations, we can explain both the existence of strong AT pattern, with C to T transition mutations dominating over C to R (purine) or A to Y (pyrimidine) transversion mutations, and also the increase in pattern strength around longer microsatellites, with interruptions in longer arrays more likely to be internal to the repeat tract and hence be excluded from the analysis. However, we suggest that this model is unlikely for two reasons. First, such a model fails to accommodate the strong asymmetry in patterning that is observed for some dinucleotides and specific cassette bases around the (AC)*_n_* repeat tract. Polarity has been noted for minisatellite mutations, with mutational processes differing between the two ends of the repeat tract (Armour et al. 1993; Jeffreys et al. 1994), but a microsatellite is much simpler in structure than a minisatellite and any polarity would have to affect some dinucleotides but not others. Second, the commonest and strongest patterning is observed for dinucleotide AT, and this would require high rates of C to T transitions but effectively no A to G transitions. More generally, the microsatellite erosion model predicts that flanking sequence patterning should be dominated by purine/pyrimidine, and this is not the case (see Table 2).

The patterning we describe appears to represent an important component of the forces that shape genome evolution, both in terms of its ubiquity and the absolute strength of its effect. It follows that there are many possible practical and theoretical implications. For example, even very short microsatellites appear able to cause some level of convergent sequence evolution, and hence to confound phylogenetic analyses. Similarly, microsatellites near genes may increase local mutation rates and influence the spectrum of new mutations that arise. To explore the size of these effects we designed experiments both to measure absolute convergence and to ask about evidence for changes in mutation rate.

To measure convergence, we made various comparisons between blocks of 50 bases chosen randomly, lying next to a microsatellite and lying near a microsatellite. We found an ordered progression of similarity from 12.77/50 bases for random–random through to a maximum of 14.31/50 bases between blocks adjacent to microsatellites 7–10 repeats long, an increase of 12% similarity. Although modest, trends are highly significant, with all comparisons showing a dependency on microsatellite length that peaks at around 7–10 repeats. The most parsimonious explanation for these similarities is that sequences flanking AC microsatellites tend to be AT-rich and to exhibit increased simplicity. Both these characteristics would increase the chance of flanking sequences being unusually similar both to each other and to random sequences that may contain polyA tails or other sources of simplicity. At the same time, the high scores gained by (AC)_7–10_ both for assignment to their own class and for similarity to each other relative to random blocks provide a clear indication that convergent sequence evolution is occurring. Interestingly, any given flanking sequence tends to be more similar to a block 500 bases away than to a similarly placed block near a different microsatellite, suggesting longer range patterning such as might arise through placement within the same isochore (Bernardi 2000). Furthermore, our attempts to measure variation in mutation rate indicate reduced similarity between homologous human and chimpanzee sequences, implying a higher rate of evolution, at least for a region in the order of ten bases around the microsatellite. On a scale of blocks of 20 bases the trends are less convincing. Having said this, it seems likely that any genuine variation in mutation rate would be to some extent masked by the convergent evolution, and hence that this aspect would benefit from further investigation.

In conclusion, previous studies of microsatellite flanking sequences have identified several features, including a tendency to harbour other microsatellites, a locally increased mutation rate, and, conversely, conservation over unexpectedly large tracts of evolutionary time. Our analyses support all these trends and provide a possible resolution for the apparent contradiction between faster evolution but at the same time greater sequence conservation. Although there is evidence that mutation rates near microsatellites are elevated, we also find evidence of convergent evolution. Consequently, the increased rate of change may be to some extent neutralised and perhaps even reversed by the tendency for similar changes to occur in related lineages. Furthermore, the greatest changes appear to occur in flanking sequences around microsatellites that are below the length used as markers, at least in humans. Overall, therefore, we have been able to formalise previous anecdotal evidence and hence to document a remarkably widespread source of directional change and nonrandom evolution that undoubtedly plays an important role in shaping the make-up of our genomes.

## Materials and Methods

### 

#### Dataset.

Our dataset of (AC)*_n_* dinucleotide repeats was extracted from the human genome (build 33, NCBI Reference Sequences; NCBI, Bethesda, Maryland, United States) using a custom macro written in Visual Basic. Only microsatellites separated by at least 100 bp from the nearest (AC)_2_ or longer were included in the dataset. Thus (AC)_2_AT(AC)_10_ would not be included in the dataset, whereas ACAT(AC)_10_ would be included as (AC)_10_. Flanking sequences are here defined as the 50 bases lying either side of a microsatellite. No attempt was made to translate TG repeats with complementary AC repeats on the opposite strand. Consequently, all our microsatellites are 5′-(AC)*_n_*-3′.

#### Flanking sequence base composition.

For each frequency estimate, 95% confidence intervals were derived based on the binomial distribution (*n* < 200 observations) or a normal approximation (*n* ≥ 200 observations). Bases used to define cassette type were excluded from all calculations, and expected frequencies were taken as the average frequency across all positions.

#### Convergence of flanking sequence pattern: assignment test.

Microsatellites were divided into 21 classes according to repeat number. Class 1 was the control set, comprising 5,000 randomly selected, non-microsatellite-associated sequences from Chromosome 1. All other classes contained flanking sequences from single-length microsatellites, except class 21, which contained combined data from microsatellites 21–25 repeats long. Analysis was restricted to the most abundant cassette class, T/A, yielding sample sizes that peaked at 1,087 for (AC)_5_ and declined to 175 for (AC)_20_ (see Figure 2).

As an index of similarity, we calculated the log likelihood of observing a given sequence based on its position-specific dinucleotide motif composition:



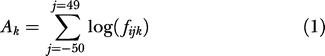



where *f_ijk_* is the frequency of dinucleotide *i* at position *j* (*j* ≠ −1 or 0, with position 0 including the microsatellite and its cassette bases) in flanking sequences of class *k*. To avoid bias, when a sequence was compared with its own class, its contribution to the dinucleotide frequencies was first removed. For each sequence in turn, *A* was calculated for every class and the sequence was then assigned to the class that yielded the highest index value. Under convergent evolution, we expect sequences to tend to be assigned to their own or similar length classes.

#### Convergence of flanking sequence pattern: quantifying sequence change.

Sequences were again divided into length classes 2 to 21, and each sequence contributed four 50-bp blocks of sequence, one from each side immediately adjacent to the microsatellite but excluding the cassette bases (class 1), and one from each side displaced by a randomly selected number 500–600 bases distal (class 2). In addition, we also generated a database of 5,000 non-microsatellite-associated sequences. When making comparisons within a class, nonindependence was avoided by randomising the sequence order and then comparing sequence 1 with sequence 2, 2 with 3, …, (n − 1) with n. Our index of similarity was simply a count of the number of matching bases. A few pairs of sequences (less than 0.1%) gave high similarity scores of over 30/50 matching bases, presumably because these loci have been duplicated or lie in repetitive elements. Such sequences were discarded. As with all other analyses, sequences containing base ambiguities (marked base N) were also discarded.

#### Rate of evolution around microsatellites.

(AC)*_n_* repeat microsatellites were extracted from the available chimpanzee finished-quality high-throughput genomic sequence (NCBI) as outlined above for humans. A 300-base region 220 bases upstream from each chimpanzee microsatellite was used by Megablast (Win32 version 2.2.6, NCBI) to identify homologous human loci in the finished genome sequence. Sequences with multiple high-scoring hits were discarded, as they presumably occur because a locus is found in repetitive elements or has been duplicated. Those nonoverlapping hits with at least 280/300 matching bases and an expectation (e-value) greater than five times that of any other hit to the same sequence were thus retained, giving a dataset of 5,537 sequences.

## Supporting Information

Figure S1 Flanking Sequence Nucleotide Frequency Distributions Illustrating Three Classes of Patterning with Neither Strong Periodicity Nor Asymmetry From little structure of any kind (A) to complicated aperiodic clustering (C). Plots are as described in Figure 3.
(1.7 MB TIF).

Figure S2 Flanking Sequence Frequency Distributions for Four Dinucleotide Motif–Cassette Combinations Further to Those Shown in Figure 4
Plots are as described in Figure 4.
(3.0 MB TIF).

Figure S3 Dinucleotide Flanking Sequence Patterning in Interspersed Repeats and Unique Sequence DNA Figure depicts equivalent patterns of asymmetry in AT dinucleotide frequencies for the commonest cassette type, (T/A), around microsatellites in unique sequence DNA (A), LINE/L1 elements (B), and SINE/Alu elements (C). Plotting conventions are the same as for Figure 4.
(1.4 MB TIF).

Figure S4 Dependence of Differences among Homologous Loci on Location of Microsatellite Block position is relative to the central microsatellite (not shown).
(A and B) Proportion of exact matches (with 95% binomial confidence intervals) and average number of matches, excluding exact matches (± standard error), with block position around (AC)_2–3_ microsatellites (*n* = 4,593).
(C and D) As (A and B) but for (AC)_4+_ microsatellites (*n* = 356). Average proportion of exact matches and number of matches, calculated separately for 5′ and 3′ blocks around (AC)_2–3_ microsatellites, are shown by a black line in (A) and (C), and (B) and (D), respectively. Average percentage match rather than average match is plotted in (B) and (D) because overlapping blocks were truncated to exclude overlapping regions from the analysis, with the result that not all blocks contained 20 bases.
(2.9 MB TIF).

Figure S5 Mean Proportion of Mismatches along Homologous Flanking Sequences The proportion of mismatches occurring at a given base in a flanking sequence are averaged over (AC)_2–3_ microsatellite loci (A) and over (AC)_4+_ microsatellite loci (B). Shown ± standard error. The microsatellite at base position 0 is not shown. Expectation, calculated separately for 5′ and 3′ sequences around (AC)_2–3_ microsatellites, is shown by a black line in both plots.
(3.4 MB TIF).

## Abbreviations

NCBI: National Center for Biotechnology Information
