# Supplementary figures and images for: Evidence for Widespread Convergent Evolution around Human Microsatellites
Source: PLoS Biol. 2004 Aug 17;2(8):e199. doi: 10.1371/journal.pbio.0020199 (PMC509290; doi:10.1371/journal.pbio.0020199)

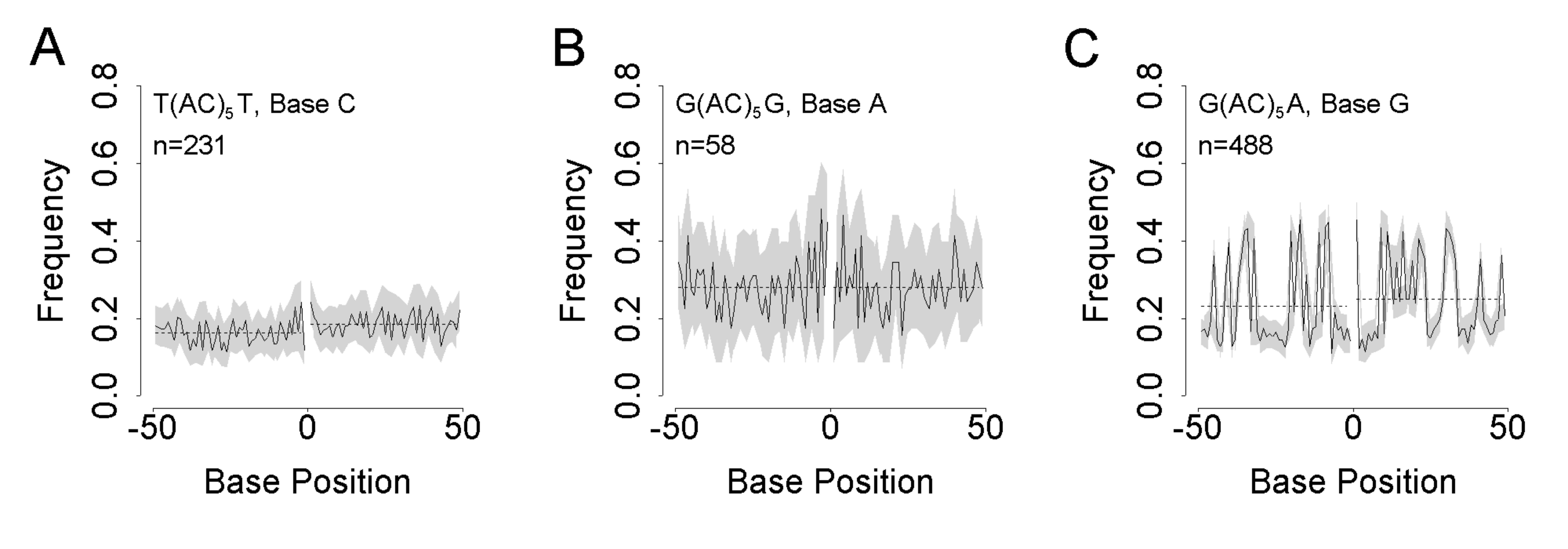

Supplement: Figure S1 — From little structure of any kind (A) to complicated aperiodic clustering (C). Plots are as described in Figure 3. (1.7 MB TIF). [file pbio.0020199.sg001.tif]

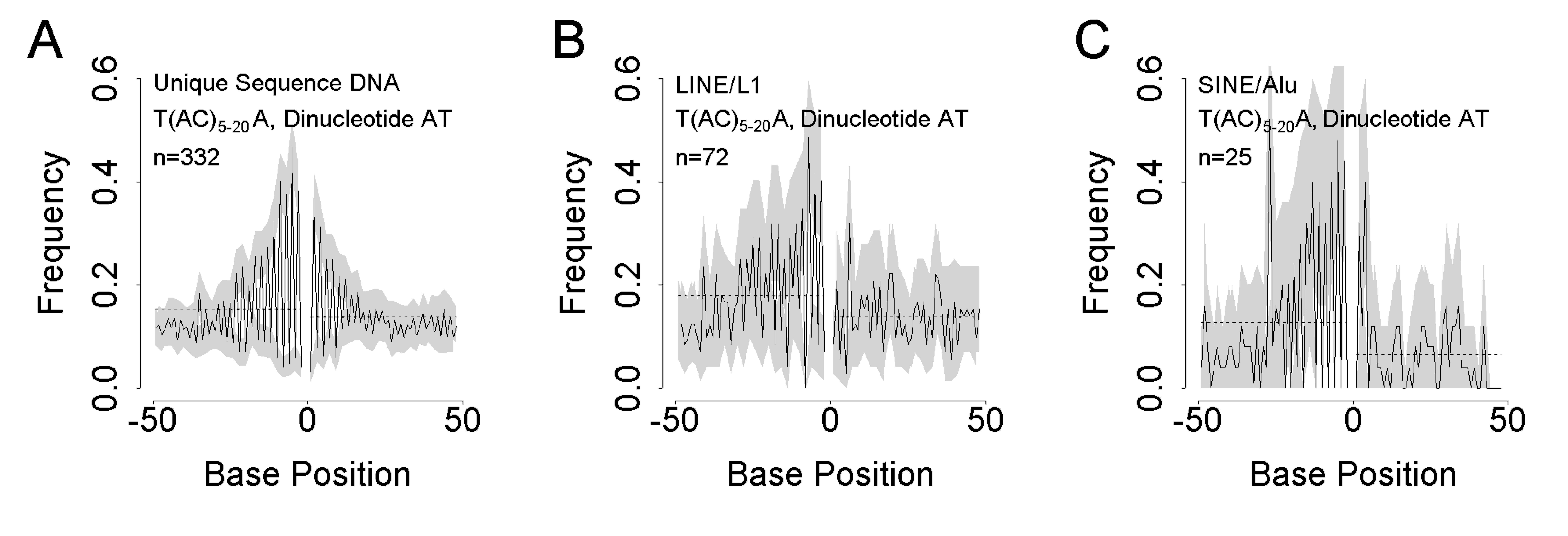

Supplement: Figure S3 — Figure depicts equivalent patterns of asymmetry in AT dinucleotide frequencies for the commonest cassette type, (T/A), around microsatellites in unique sequence DNA (A), LINE/L1 elements (B), and SINE/Alu elements (C). Plotting conventions are the same as for Figure 4. (1.4 MB TIF). [file pbio.0020199.sg003.tif]

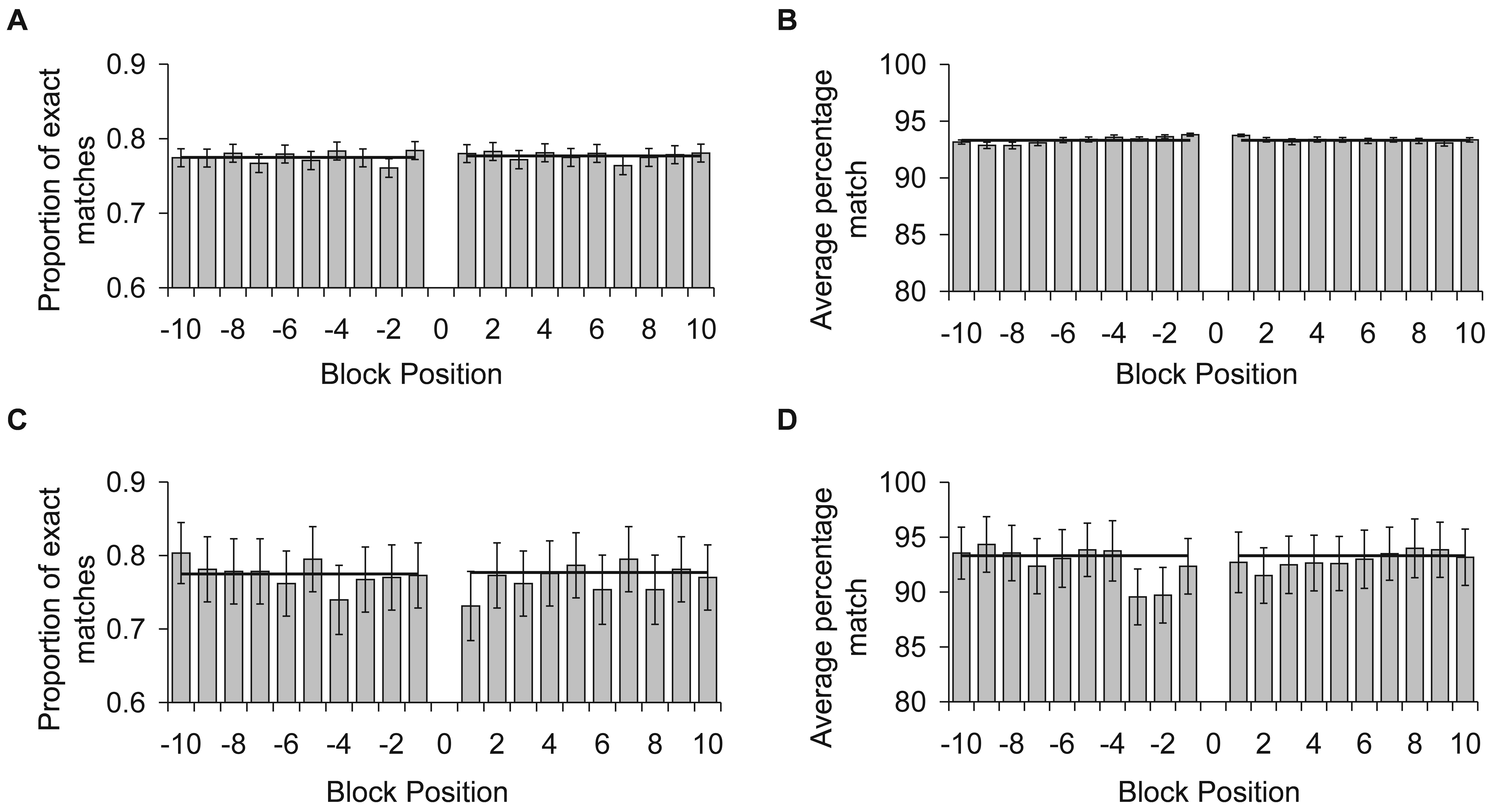

Supplement: Figure S4 — Block position is relative to the central microsatellite (not shown). (A and B) Proportion of exact matches (with 95% binomial confidence intervals) and average number of matches, excluding exact matches (± standard error), with block position around (AC)2–3 microsatellites (n = 4,593). (C and D) As (A and B) but for (AC)4+ microsatellites (n = 356). Average proportion of exact matches and number of matches, calculated separately for 5′ and 3′ blocks around (AC)2–3 microsatellites, are shown by a black line in (A) and (C), and (B) and (D), respectively. Average percentage match rather than average match is plotted in (B) and (D) because overlapping blocks were truncated to exclude overlapping regions from the analysis, with the result that not all blocks contained 20 bases. (2.9 MB TIF). [file pbio.0020199.sg004.tif]
